# Honokiol Protected against Heatstroke-Induced Oxidative Stress and Inflammation in Diabetic Rats

**DOI:** 10.1155/2014/134575

**Published:** 2014-02-17

**Authors:** Chuan-Chih Hsu, Li-Fan Chen, Mao-Tsun Lin, Yu-Feng Tian

**Affiliations:** ^1^Department of Surgery, School of Medicine, College of Medicine, Taipei Medical University, Taipei 112, Taiwan; ^2^Division of Cardiovascular Surgery, Department of Surgery, Taipei Medical University Hospital, Taipei 112, Taiwan; ^3^Nursing Department, Cheng Kung University Hospital, Tainan 701, Taiwan; ^4^Department of Nursing, Chang Jung Christian University, Tainan 712, Taiwan; ^5^Department of Medical Research, Chi Mei Medical Center, Tainan 710, Taiwan; ^6^Department of Surgery, Chi Mei Medical Center, Tainan 710, Taiwan; ^7^Department of Health and Nutrition, Chia Nan University of Pharmacy and Science, Tainan 712, Taiwan

## Abstract

We aimed at investigating the effect of honokiol on heatstroke in an experimental rat model. Sprogue-Dawley rats were divided into 3 groups: normothermic diabetic rats treated with vehicle solution (NTDR+V), heatstroke-diabetic rats treated with vehicle (HSDR+V), and heatstroke rats treated with konokiol (0.5–5 mg/ml/kg) (HSDR+H). Sixty minutes before the start of heat stress, honokiol or vehicle solution was administered. (HSDR+H) significantly (a) attenuated hyperthermia, hypotension and hypothalamic ischemia, hypoxia, and neuronal apoptosis; (b) reduced the plasma index of the toxic oxidizing radicals; (c) diminished the indices of hepatic and renal dysfunction; (d) attenuated the plasma systemic inflammatory response molecules; (e) promoted plasma levels of an anti-inflammatory cytokine; (f) reduced the index of infiltration of polymorphonuclear neutrophils in the serum; and (g) promoted the survival time fourfold compared with the (HSDR+V) group. In conclusion, honokiol protected against the outcome of heatstroke by reducing inflammation and oxidative stress-mediated multiple organ dysfunction in diabetic rats.

## 1. Introduction

Among the patients with heatstroke at the Mecca Pilgrimage, most of them had diabetes with hyperglycemia [1]. Indeed, compared with nondiabetic rats, diabetic rats were more susceptible to heat stroke occurrence [2, 3]. Most of the heatstroke manifestations (e.g., excessive hyperthermia associated with a systemic inflammatory response that led to multiple organ dysfunction in which the brain dysfunctions predominated) could be reproduced by exposing the streptozotocin-induced diabetic rats to hot environments [4]. The heatstroke-diabetic rats (HSDR) displayed excessive hyperthermia, brain inflammation, ischemia and oxidative damage, and multiple organ dysfunction or failure.

Honokiol was one of main constituents of the Chinese herb, Magnolia officinalis, and had a variety of pharmacological actions, including anti-inflammatory effects [5], antimicrobial activity [6, 7], and antioxidative effects [8]. However, it was not known whether hypothalamic ischemia and oxidative stress with systemic inflammation and multiple organ dysfunction in HSDR could be affected by honokiol.

Heatstroke was a form of hyperthermia associated with a systemic inflammatory response that led to multiple organ dysfunction, in which central nervous system (CNS) disorders predominated [2]. Based on triad of factors (hyperthermia, CNS disorders, and a history of heat stress), anesthetized rodents all displayed a uniform response which was similar to responses of humans with heatstroke [3].

The present study was to assess the effects of heat stress on body core temperature (*T*_co⁡_), mean arterial pressure (MAP), hypothalamic values of cerebral blood flow (CBF), partial pressure of O_2_ (PO_2_), cellular ischemia marker (e.g., glutamate), organ damage markers (e.g., glycerol and lactate dehydrogenase), toxic oxidizing radicals (e.g., nitric oxide and dihydroxybenzoic acid), and serum levels of inflammatory markers (e.g., interleukin-1*β*, interleukin-6, tumor necrosis factor-*α*, and myeloperoxidase) in HSDR with or without honokiol pretreatment.

## 2. Methods

### 2.1. Animals

Adult male Sprague-Dawley rats (weighing 223 to 256 g) were obtained from the Animal Resource Center of the National Science Council of the Republic of China (Taipei, Taiwan). The animals which were allowed to become acclimated for at least one week were housed four per cage at an ambient temperature (*T*_*a*_) of 22 ± 1°C with a 12-h light/dark cycle and were supplied with rat chow and tap water *ad libitum*. The experimental protocol was approved by the Animal Ethic Committee of the Chi Mei Medical Center (Tainan, Taiwan) under guidelines of the National Science Council. Animal care and experiments were according to the Guide for the Care and Use of Laboratory Animals published by the USA National Institutes of Health (NIH publication number 85-23 revised 1996). Adequate anesthesia was maintained to abolish the corneal reflex and pain reflexes induced by tail pinching throughout all experiments (approximately 480 minutes in duration) by a single intraperitoneal dose of urethane (1.4 g/kg body weight). At the end of the experiments, control rats and all rats that had survived heat stroke were sacrificed with an overdose of urethane.

### 2.2. Surgery and Physiological Parameter Monitoring

The right femoral artery and vein of rats under urethane anesthesia were cannulated with polyethylene tubing (PE50), for blood pressure monitoring and drug administration. The *T*_co⁡_ temperature (*T*_co⁡_) was monitored continuously by means of a thermocouple, while MAP was monitored continuously with a pressure transducer.

### 2.3. Induction of Diabetes

Diabetes was induced by injecting streptozotocin (Sigma, St. Louis, MO, USA) at 30 mg/mL/kg of body weight in the tail veins of unanesthetized rats. The animals were maintained for 4-5 weeks before heat stress was applied. At the day of thermal experiments, the right femoral artery and vein of rats under general anesthesia were cannulated with polyethylene tubing (PE50) for blood pressure monitoring and drug administration or biochemical determination, respectively.

### 2.4. Introduction of Heatstroke

**Figure 1:**
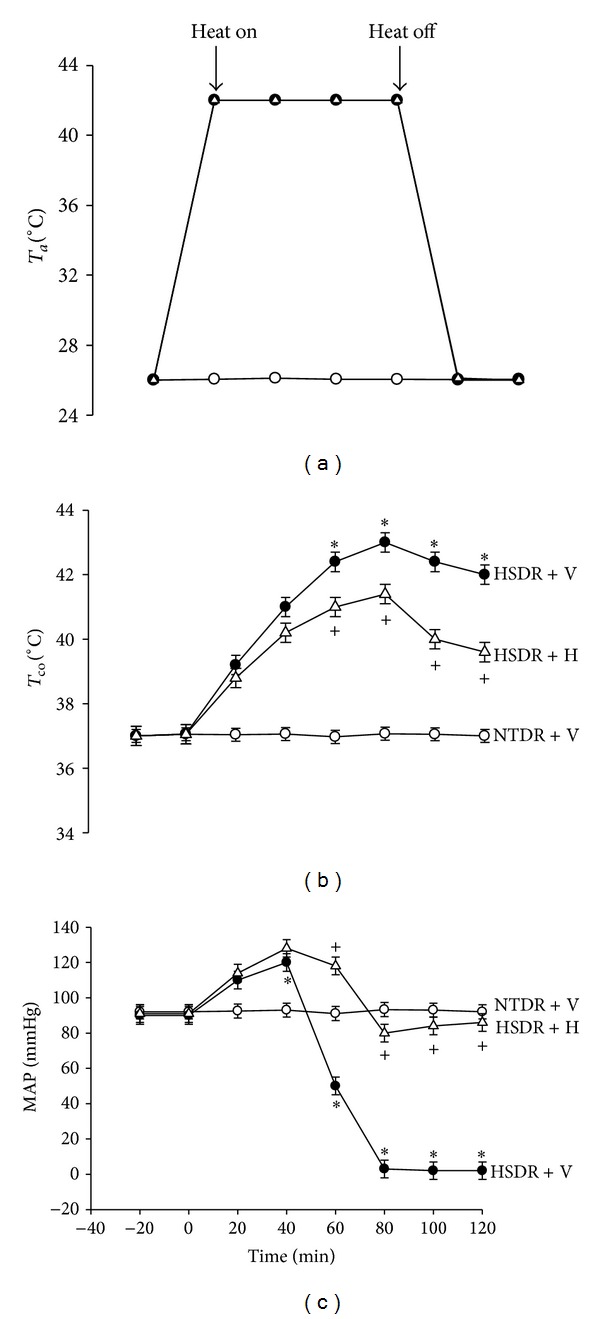
Values of both body core temperature (*T*_co⁡_) and mean arterial pressure (MAP) during different ambient temperatures (*T*_*a*_) for normothermic diabetic rats treated with vehicle solution (NTDR + V; ○), heatstroke-diabetic rats treated with vehicle solution (HSDR + V; ●), and heatstroke-diabetic rats treated with honokiol (HSDR + H; △). Data were means ± standard deviation (SD) of eight animals per group. *P < 0.05, in comparison with the (○) group; ^+^ *P* < 0.05, in comparison with the (●) group.

In the present study, the core temperature (at about 37°C) of the anesthetized animals was maintained with an infrared light lamp except in the heat stress experiments. Heatstroke was induced by putting the animals in a bold heating pad of 43°C controlled by circulating hot water. The instant in which MAP dropped to ~50 mmHg was found to be about 60 minutes after the start of heat stress (Figure 1). At 60 minutes, the heating was removed and the animals were allowed to recover at room temperature (26°C). Survival time values (interval between the start of heat stress and animal death) were determined. It was seen in Figure 1 that the heated rats displayed both hyperthermia (~42.5°C) and hypotension (~50 mmHg) at 60 minutes, suggesting the occurrence of heatstroke [3].

### 2.5. Experimental Groups

Animals were assigned randomly to one of the following three major groups: normothermic diabetic rats treated with vehicle solution (NTDR + V), heatstroke-diabetic rats treated with vehicle solution (HSDR + V), and heatstroke-diabetic rate treated with honokiol (HSDR + H). Sixty minutes before the start of heat stress, honokiol (H_8_C_2_O_2_) (0.5 mg, 1.5 mg, or 5.0 mg per mL per kg of body weight; Sigma-Aldrich, Saint Louis, MO, USA) or vehicle (DMSO) solution (1 mL per kg body weight) was administered via the veins.

### 2.6. Measurement of Cerebral Blood Flow (CBF) and Partial Pressure of Oxygen (PO_2_) in the Hypothalamus

A 100 *μ*m diameter thermocouple and two 230 *μ*m fibers were attached to the oxygen probe. This combined probe measured oxygen, temperature, and microvascular blood flow. The measurement required OxyLite and OxyFlow instruments. OxyLite 2000 (Oxford Optronix Ltd., Oxford, UK) was a 2-channel device (measuring PO_2_ and temperature at two sites simultaneously), whereas OxyFlo 2000 was a 2-channel Laser Doppler perfusion monitoring instrument. This combined probe was implanted stereotaxically into the right hypothalamus according to the stereotaxic coordinates of Paxinos and Watson [9] to measure both CBF and PO_2_ in the hypothalamus.

### 2.7. Determination of Serum Levels of the Toxic Oxidizing Radicals

For determination of NO_*x*_^−^ and DHBA, blood samples were taken 0 and 60 minutes after the start of heat exposure. The NO_*x*_^−^ concentrations in the dialysates were measured with the Eicomo-20 NO_*x*_^−^ analysis system (Eicom, Kyoto) [10]. The concentrations of hydroxyl radicals were measured by a modified procedure based on the hydroxylation of sodium salicylates by hydroxyl radicals, leading to the production of 2,3-DHBA and 2,5-DHBA [11].

### 2.8. Quantification of Organ Function and Injury

For determination of creatinine, blood urea nitrogen (BUN), alanine aminotransferase (ALT), aspartate aminotransferase (AST), alkaline phosphatase (ALP), and lactate dehydrogenase (LDH) were estimated in blood samples collected at 0 and 60 minutes after the start of heat stress. The serum levels of creatinine, BUN, ALT, AST, and ALP were determined by spectrophotometry (HITACHI7600, Tokyo, Japan). In addition, LDH was measured to evaluate the extent of organ injury by Fuji DRI-CHEM 3030 (Fuji Photo Film Co., Ltd., Tokyo, Japan).

### 2.9. Measurement of Serum IL-1*β*, IL-6, TNF-*α*, IL-10, and ICAM-1 Levels

Blood samples were collected 0 and 60 minutes after the start of heat stress and stored at −80°C until they could be assayed. We used commercially available ELISA kits for the determination of serum IL-1*β*, IL-6, TNF-*α*, IL-10, and ICAM-1 levels (Quantikine, R&D Systems Inc., Minneapolis, MN, USA) according to the manufacturer's instruction.

### 2.10. Measurement of E-Selectin

Peripheral polymorphonuclear (PMN) cells were isolated from the whole blood of rats and treated with heparin (100 units/mL). Erythrocytes were allowed to sediment for 30 minutes after the addition of 3 mL of 6% dextran (weight/volume in PBS) to 10 mL blood. After sedimentation, the plasma containing leukocytes was centrifuged twice at 300 g for 5 min each. The precipitates were mixed with 70% osmolality-adjusted Percoll and centrifuged at 30,000 g for 30 min at 26°C. The PMN-riched layer was fractionated. Each fraction was washed twice with Hanks' balanced salt solution, and the cell number was counted. The purity of the PMNS was determined to exceed 95% by Giemsa Staining Cells (1 × 10^6^ cells/tube) which were incubated with a rabbit polyclonal antibody to (CD62E (ab18981; Abcam PIC332, Cambridge, UK) or control. After washing, the cells were stained with a secondary antibody (goat polyclonal to rabbit IgG-H & L [FITC] • [ab6717]; Abcam PIC). Cells were incubated for 1 hour at 4°C and washed. They were mixed with oligasaccharides and incubated for 20 minutes, and then coincubated with KM93 for 60 minutes. The fluorescence intensity of cells was analyzed with a FACStar (Becton Dickinson).

### 2.11. Determination of Myeloperoxidase (MPO) Activity

A spectrophotometric method [12] was used to determine MPO activity in the serum. A 100 *μ*L aliquot of serum was mixed with 900 *μ*L of 50 mmol/L phosphate buffer (pH-6.0) containing 0.167 mg/mL of o-dianisidine dihydrochloride and 0.0005% hydrogen peroxide. One unit of peroxidase activity equaled the amount of enzyme decomposing 1 *μ*mol of hydrogen peroxide which was calculated from the oxidation of o-dianisidine using an absorption coefficient of 11.3/mM/cm at 460 nm.

### 2.12. Assessment of Plasma Corticosterone and ACTH

Plasma corticosterone and ACTH were assayed using Corticosterone Double Antibody RIA kit (MP Biomedicals, Solon, Oh, USA) and ACTH (Rat, Mouse)-RIA kit (Phoenix Pharmaceuticals, Burlingame, CA, USA), respectively. All analyses were performed according to manufacturer's instruction.

### 2.13. Statistical Analysis

All data were expressed as means ± standard deviation. One-way analysis of variance with Tukey's multiple comparisons test was used for serum markers and physiological parameters. Significant differences were established at *P* < 0.05. For all statistical analyses, SPSS soft ware version 10.0 (SPSS Inc., Chicago, IL, USA) was used.

## 3. Results

Diabetic rats had smaller body weight, higher plasma glucose levels, and lower plasma insulin when compared to healthy rats (Table 1).

The survival time values for (HSDR + V) rats were decreased from the (NTDR + V) control values of 480 ± 3 min to new values of 79 ± 3 min after the start of heat stress (Table 2). Heatstroke-diabetic rats (HSDR) treated with H (0.5, 1.5, and 5.0 mg/mL/kg of honokiol) 60 minutes before heat stress had significantly and dose-dependently higher values of survival time (114–273 min) than those treated with vehicle. However, in the following experiments, only the effects of one dose of 1.5 mg/mL/kg of honokiol were on determining other parameters during heatstroke.

Figure 1 showed the effects of heat exposure (43°C for 60 minutes) on both *T*_co⁡_ and MAP in (NTDR + V) rats, (HSDR + V) rats, and (HSDR + H) rats. As shown in this figure, sixty minutes after the start of heat exposure, the values of MAP in the (HSDR + V) group were significantly lower than those in the (NTDR + V) group (~51 mmHg versus ~92 mmHg; *P* < 0.05). On the other hand, the values of *T*_co⁡_ in the (HSDR + V) group were significantly higher than those in the (NTDR + V) group (~42.2°C versus ~37.3°C; *P* < 0.01). Heat-induced hypotension and hyperthermia were all significantly reduced by honokiol pretreatment as shown in (HSDR + H) group.

**Figure 2:**
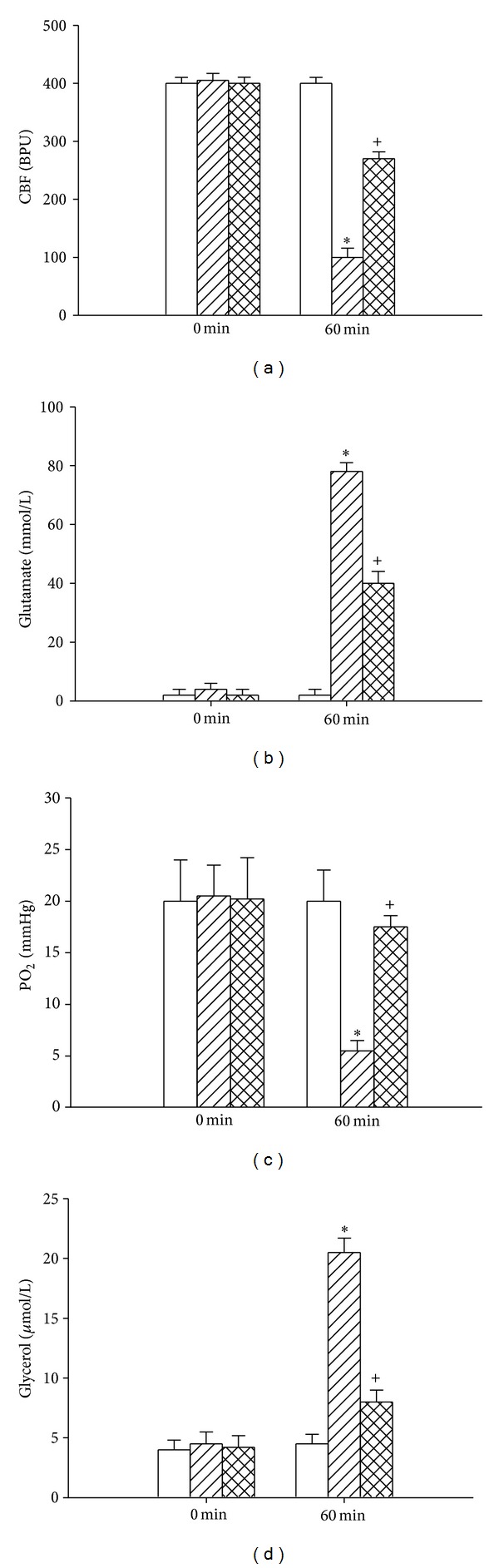
Values of hypothalamic cerebral blood flow (CBF), brain partial pressure of O_2_ (PO_2_), and cellular levels of glutamate and glycerol for the (NTDR + V) group (white bar), (HSDR + V) group (dashed bar), and (HSDR + H) group (crossed bar). The values were obtained at 0 or 60 min after the initiation of heat exposure in heatstroke rats or the equivalent times in the (NTDR + V) group. *P < 0.01 in comparison with the (white bar) group; ^+^^+^ *P* < 0.05 in comparison with the (dashed bar) group. All heatstroke groups had heat exposure (43°C) withdrawn exactly at 60 min and were then allowed to recover at room temperature (26°C). Bars were the mean ± SD of eight rats for each group. Please see the legends of Figure 1 for explanation of the abbreviations.

Figure 2 showed that the hypothalamic levels of PO_2_ and cerebral blood flow in the (HSDR + V) rats were significantly lower at 60 minutes after the start of heat exposure than in the (NTDR + V) group (~100 BPU versus ~400 BPU for CBF; ~5 mmHg versus ~20 mmHg for PO_2_; *P* < 0.05). In contrast, the hypothalamic levels of glutamate and glycerol in the (HSDR + V) rats were significantly higher at 60 minutes after the start of heat exposure (~82 mmol/L versus ~3 mmHg for glutamate; ~22 *μ*mol versus ~5 *μ*mol for glycerol; *P* < 0.05). Heat-induced hypothalamic hypoxia, ischemia, and damage at 60 minutes were all significantly reduced by pretreatment with honokiol.

**Figure 3:**
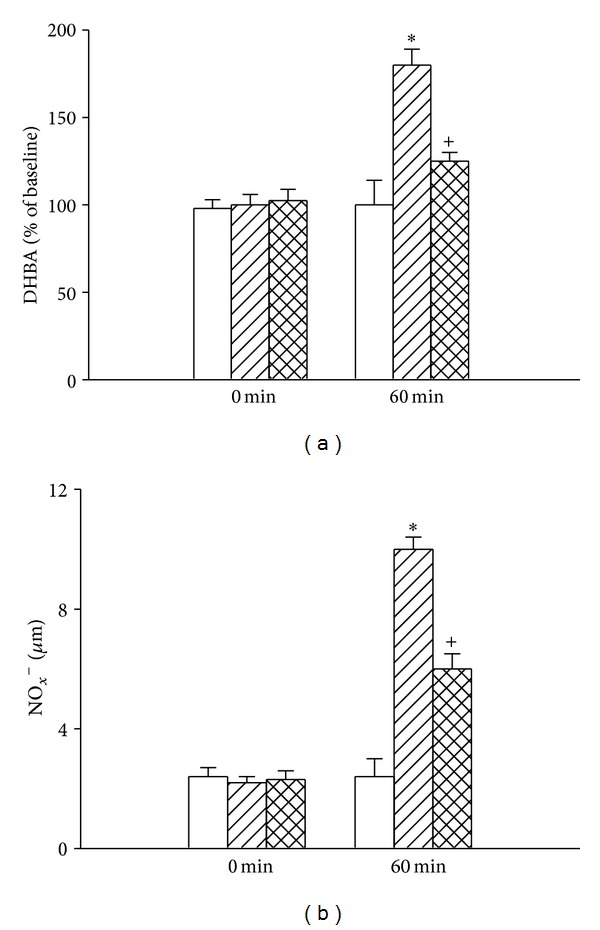
Values of hypothalamic levels of dihydroxybenzoic acid (DHBA) and nitric oxide metabolites (NO_*x*_^−^) for the (NTDR + V) group (white bar), (HSDR + V) group (dashed bar), and (HSDR + H) group (crossed bar). The values were obtained at 0 or 60 min after the initiation of heat exposure in heatstroke rats or the equivalent times in the (NTDR + V) group. *P < 0.01 in comparison with the (white bar) group; ^+^ *P* < 0.05 in comparison with the (dashed bar) group. Bars were the mean ± SD of eight rats for each group. See the legends of Figure 1 for explanation of the abbreviations.

Figure 3 showed that the hypothalamic levels of 2,3-DHBA and NO_*x*_^−^ in the (HSDR + V) rats were significantly higher at 60 minutes after the start of heat exposure than in the (NTDR + V) rats (~180% versus ~100 mmHg for DHBA; ~9.6 *μ*M versus ~2.6 *μ*M for NO_*x*_^−^; *P* < 0.05). As shown in this figure, heat-induced increased hypothalamic levels of all these parameters were significantly reduced by honokiol pretreatment in the (HSDR + H) rats.

**Figure 4:**
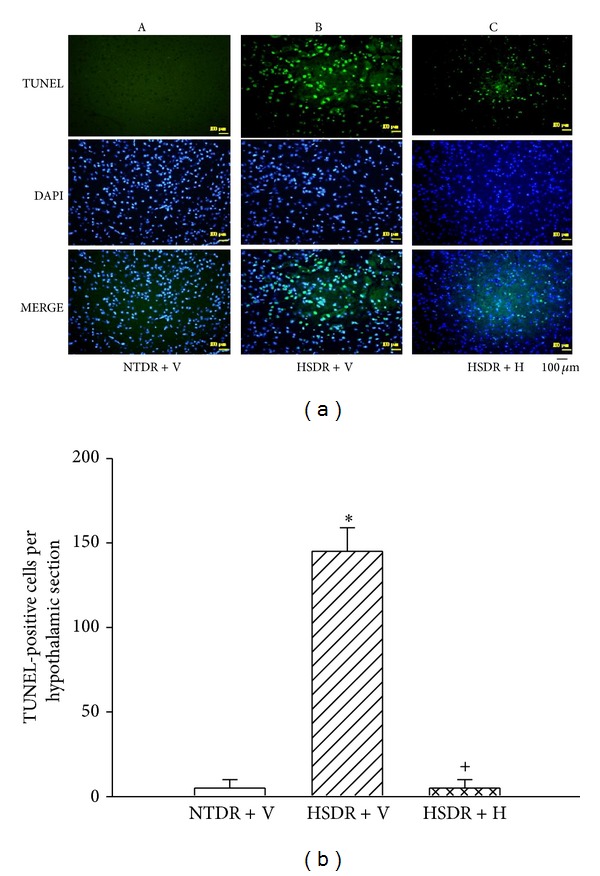
Values of hypothalamic TUNEL-positive cells for the (NTDR + V) group (white bar), (HSDR + V) group (dashed bar), and (HSDR + H) group (crossed bar). The values were obtained at 60 min after the initiation of heat exposure in heatstroke rats or the equivalent times in the (NTDR + V) group. *P < 0.01 in comparison with the (white bar) group; ^+^ *P* < 0.01 in comparison with (dashed bar) group. Bars were each the mean ± SD of eight rats of reach group. Top panels depict the representative photographs for TUNEL staining in a (NTDR + V) rat, a (HSDR + V) rat, and a (HSDR + H) rat. See the legends of Figure 1 for the abbreviation.

Figure 4 showed that the hypothalamic values of TUNEL-positive cells in the (HSDR + V) were significantly higher at 60 minutes after the start of heat stress than that in the (NTDR + V) rats (~148 cells versus ~5 cells; *P* < 0.01). As shown in this figure, heat-induced increased hypothalamic values of TUNEL-positive cells were significantly reduced by honokiol pretreatment in the (HSDR + H) rats.

**Figure 5:**
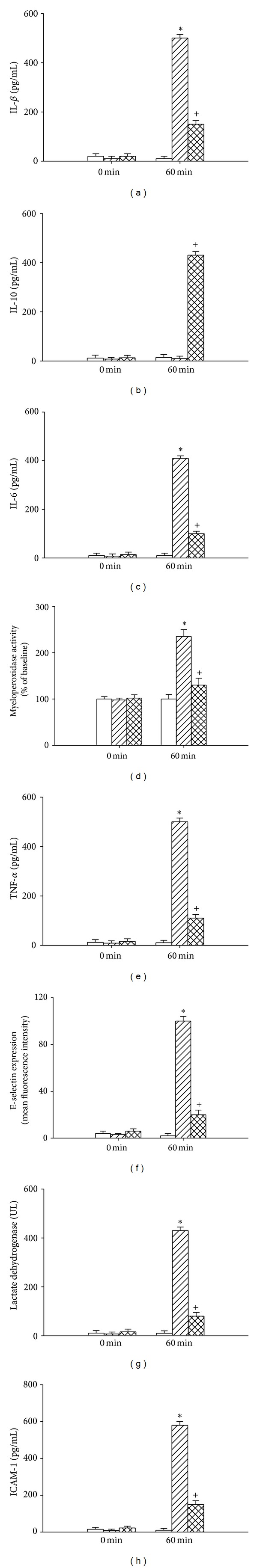
Values of serum interleukin-1*β* (IL-*β*), IL-6, tumor necrosis factor-*α* (TNF-*α*), E-selectin, ICAM-1, lactate dehydrogenase, and myeloperoxidase (MPO) for the (NTDR + V) group (white bar), (HSDR + V) group (dashed bar), and (HSDR + H) group (crossed bar). The values obtained at 0 or 60 min after the initiation of heat exposure in heatstroke rats or the equivalent times in the (NTDR + V) group. *P < 0.01 in comparison with the (white bar) group; ^+^ *P* < 0.05 in comparison with the (dashed bar) group. Bars were the mean ± SD of eight rats for each group.

Figure 5 showed that the serum values of IL-1*β*, IL-6, TNF-*α*, E-selectin, ICAM-1, lactate dehydrogenase, and MPO for (HSDR + V) were significantly higher at 60 minutes after the start of heat stress than that in the (NTDR + V) rats. Heat-induced increased values of these parameters were all significantly reduced by honokiol pretreatment in the (HSDR + H) rats. In addition, serum levels of IL-10 in the (HSDR + H) rats were significantly higher than that in the (HSDR + V) rats at 60 minutes after the start of heat stress.

Table 3 showed that the plasma levels of both ACTH and corticosterone in the (HSDR + V) rats were significantly higher at 60 minutes after the start of heat stress than that in the (NTDR + V) rats. Additionally, (HSDR + H) rats had significantly (*P* < 0.05) higher plasma levels of both ACTH and corticosterone than (HSDR + V) rats.

Table 4 showed that the serum level of BUN, creatinine ALT, AST, and AP in the (HSDR + V) rats were significantly higher at 60 min after the start of heat stress than that in the (NTDR + V) rats. Heat-increased serum levels of BUN, creatinine, ALT, AST, and AP in the (HSDR + V) rats were significantly reduced by honokiol pretreatment in the (HSDR + H) rats.

## 4. Discussion

Severe heat stress induced excessive hyperthermia, splanchnic vasoconstriction, and multiple organ dysfunction or failure. During heatstroke, excessive hyperthermia facilitates the leakage of endotoxin from the intestine to the circulating blood and resulted in leukocytes infiltration and excessive activation of endothelial cells [2, 13]. Tissue ischemia, hypoxia, and damage secreted proinflammatory cytokines which led to excessive production or release of the toxic oxidizing radicals like NO_*x*_^−^ and DHBA and resulted in multiple organ damage [14]. Amplification of the resultant activated inflammatory state induced accumulation of free radicals and resulted in augmentation of local tissue injury [15]. This systemic inflammation response led to polymorphonuclear cells sequestration in both the lung and liver and resulted in multiple organ dysfunction or failure [16]. In particular, heat-induced multiple organ dysfunction might be associated with hypothalamo-pituitary-adrenocortical (HPA) axis impairment [17]. Their data showed that intolerance to heat exposure was associated with an HPA axis impairment, possibly related to changes occurring in the inflammatory mediators levels.

As mentioned in Section 1, the patients with diabetes were susceptible to heat stroke at Mecca Pilgrimage [1]. Our present study further showed that heat-induced multiple organ dysfunction or failure in streptozotocin-induced diabetic rats was related to tissue inflammation and oxidative stress. Especially, pretreatment with honokiol significantly (a) attenuated hyperthermia, hypotension and hypothalamic ischemia, hypoxia, and neuronal apoptosis, (b) reduced the plasma index of the toxic oxidizing radicals including NO_*x*_^−^ and 2,3-DHBA, (c) diminished the indices of hepatic and renal dysfunction including creatinine, blood urea nitrogen, alanine aminotransferase, aspartate aminotransferase, alkaline phosphatase, and lactate hydrogenase, (d) attenuated the plasma systemic inflammatory response molecules like soluble intercellular adhesion molecule-1, E-selectin, tumor necrosis factor-alpha, interleukin-1*β*, and interleukin-6, (e) promoted plasma levels of an anti-inflammatory cytokine, interleukin-10, (f) reduced an index of infiltration of polymorphonuclear neutrophils in the serum as measured by myeloperoxidase activity, (g) enhanced the plasma levels of corticosterone and adrenocorticotrophic hormone, and (h) promoted the survival time fourfold in HSDR. Hence, we suggested that isolating honokiol from Magnolia officinalis might be a potential adjunct in the prevention of heatstroke. In fact, the biological activities of honokiol include potent inhibition of lipid peroxidation [8, 18], scavengers of hydroxyl radicals [19], inhibition of leukotriene synthesis in leukaemia cells [20], and inhibition of UV-induced mutations [21].

Serum molecules like IL-1*β*, IL-6, TNF-*α*, ICAM-1, and E-selectin were involved in the pathophysiology of systemic inflammatory response syndromes [22–25]. Activation of both neutrophils and endothelial cells is associated with overproduction of these systemic inflammatory response syndrome molecules in patients [2] or rats [26] with heatstroke. In the present study, both hyperthermia and overproduction of these systemic inflammatory response syndrome molecules in rats could be significantly attenuated by honokiol. In addition, honokiol pretreatment facilitated the production of serum IL-10, an anti-inflammatory cytokine, [27] that occurred during heatstroke. Thus, honokiol might downregulate the extent of activated inflammation via attenuating the heatstroke-induced excessive hyperthermia. The severity of heat illness was believed to depend on the degree of hyperthermia and its duration [28].

It was promoted that decreased heat tolerance was associated with HPA axis impairment [17]. As shown in the present study, hypothalamic ischemia, hypoxia, and neuronal apoptosis were associated with HPA impairment (evidenced by insufficient release of both ACTH and corticosterone) in HSDR which could be significantly reversed by honokiol. It was likely that honokiol might increase heat tolerance by reducing HPA axis impairment (as reflected by producing more ACTH and corticosterone in plasma in response to heat stress) in rats. The hypothesis is supported by our previous finding showing that glucocorticoids reduced interleukin-1 concentration and resulted in neuroprotective effects in rat heatstroke [29].

It should be mentioned that the normal rats should release more ACTH and corticosterone in response to heat stress than the normothermic controls (nonetheless, the data are not reported here). However, the plasma levels of both ACTH and corticosterone in heated diabetic rats should be lower than that of heated nondiabetic rats. The reduced release of both ACTH and corticosterone displayed by heated diabetic rats could be reversed by honokiol supplement.

## 5. Conclusion

In summary, we demonstrated that honokiol protected against hyperthermia, hypotension, and multiorgan injury in HSDR. The beneficial effects of honokiol might be on the reduction of (a) systemic inflammatory response syndrome molecules like TNF-*α*, IL-1*β*IL-6, ICAM-1, MPO, and E-selectin in the plasma, (b) the toxic oxidizing radicals like NO_*x*_^−^ and 2,3-DHBA, in the plasma, (c) the organ injury indicators like LDH, creatinine, blood urea nitrogen, alanine aminotransferase, aspartate aminotransferase, and alkaline phosphatase in the plasma, and (d) an indicator for leukocytes accumulation like MPO in the serum, and thus leading to maintenance of HPA axis function and increased survival in rats with heatstroke-associated multiple organ dysfunction. Our data suggested that honokiol was useful in reducing both inflammation and oxidative stress; lowering heat-induced multiple organ dysfunction.

## Figures and Tables

**Table 1:** Body weight, plasma glucose, and plasma insulin concentration in normal and streptozotocin-induced diabetic rats.

Groups of animals	Body weight (g)	Plasma glucose (mg/dL)	Plasma insulin (µU/mL)
Normal rats	301 ± 7 (8)	213 ± 8 (8)	52 ± 6 (8)
Diabetes	256 ± 9 (8)	465 ± 17* (8)	10 ± 4* (8)

*P < 0.01 compared to normal rats.

**Table 2:** The survival time values for normothermic diabetic rats (NTDR) treated with vehicle (NTDR + V), heatstroke-diabetic rats treated with vehicle (HSDR + V), and heatstroke-diabetic rats treated with honokiol (HSDR + H).

Treatment groups	Survival time (min)
(1) NTDR + V	480 ± 3 (8)
(2) HSDR + V	79 ± 3 (8)*
(3) HSDR + H (0.5 mg/mL/kg)	114 ± 5 (8)^+ ^
(4) HSDR + H (1.5 mg/mL/kg)	186 ± 9 (8)^+ ^
(5) HSDR + H (5.0 mg/mL/kg)	273 ± 15 (8)^+ ^

All heatstroke rats which had heat exposure (43°C) were withdrawn exactly at 60 minutes and then allowed to recover at room temperature (26°C). Data are mean ± SD, followed by number of animals in parentheses. Normothermic controls (NTDR + V) were killed about 480 minutes after experiment (or at the experimental end) with the urethane overdose.

*P < 0.01, in comparison with Group 1.

^+^ *P* < 0.05, in comparison with Group 2 (Dunn's test followed by Kruskal-Wallis test). Vehicle solution or melatonin was adopted 60 minutes before the start of heat stress.

**Table 3:** The plasma levels of both adrenocorticotrophic-hormone (ACTH) and corticosterone for (NTDR + V) rats, (HSDR + V) rats, and (HSDR + H) rats.

Treatment groups/time	ACTH (pg·mL^−1^)	Corticosterone (ng·mL^−1^)
(1) NTDR + V:		
0 min	412 ± 108 (8)	139 ± 25 (8)
60 min	429 ± 113 (8)	145 ± 23 (8)
(2) HSDR + V:		
0 min	407 ± 99 (8)	128 ± 21 (8)
60 min	1063 ± 164* (8)	428 ± 23* (8)
(3) HSDR + H:		
0 min	421 ± 120 (8)	133 ± 24 (8)
60 min	1965 ± 185^+^ (8)	747 ± 33^+^ (8)

All heatstroke rats which had heat exposure (43°C) were withdrawn exactly at 60 minutes and then allowed to recover at room temperature (26°C). Data are man ± SD, followed by number of animals in parentheses.

*P < 0.05, in comparison with Group 1.

^+^ *P* < 0.05, in comparison with Group 2 (Dunn's test followed by Kruskal-Wallis test); vehicle solution (V) or honokiol (H) was adopted 60 min before the start of heat exposure.

**Table 4:** The serum levels of blood urea nitrogen (BUN), creatinine (C), alanine aminotransferase (ALT), aspartate aminotransferase (AST) and alkaline phosphate (AP) for (NTDR + V) group, (HSDR + V) group, and (HSDR + H) group.

Treatment groups/time	BUN (mmol/L)	C (mmol/L)	ALT (U/L)	AST (U/L)	AP (U/L)
(1) NTDR + V:					
0 min	8.4 ± 0.7 (8)	28 ± 2 (8)	36 ± 3 (8)	112 ± 9 (8)	308 ± 22 (8)
60 min	8.6 ± 0.6 (8)	26 ± 3 (8)	34 ± 4 (8)	115 ± 7 (8)	314 ± 24 (8)
(2) HSDR + V:					
0 min	8.6 ± 0.8 (8)	26 ± 3 (8)	35 ± 4 (8)	114 ± 8 (8)	301 ± 21 (8)
60 min	22 ± 0.9* (8)	72 ± 4* (8)	135 ± 6* (8)	556 ± 7* (8)	583 ± 27* (8)
(3) HSDR + H:					
0 min	8.5 ± 0.7 (8)	25 ± 4 (8)	32 ± 4 (8)	109 ± 8 (8)	299 ± 24 (8)
60 min	11 ± 1.1^+^ (8)	48 ± 5^+^ (8)	79 ± 5^+^ (8)	196 ± 6^+^ (8)	402 ± 19^+^ (8)

All heatstroke rats which had heat exposure (43°C) were withdrawn exactly at 60 minutes and then allowed to recover at room temperature (26°C). Data are mean ± SD, followed by number of animals in parentheses.

*P < 0.01, in comparison with Group 1.

^+^ *P* < 0.05, in comparison with Group 2 (Dunn's test followed by Kruskal-Wallis test). Vehicle solution (V) or honokiol (H) was adopted 60 min before the start of heat stress.
